# The Influence of Post-Traumatic Growth on College Students’ Creativity During the COVID-19 Pandemic: The Mediating Role of General Self-Efficacy and the Moderating Role of Deliberate Rumination

**DOI:** 10.3389/fpsyg.2021.665973

**Published:** 2021-04-14

**Authors:** Wei Zeng, Yuqing Zeng, Yanhua Xu, Dongtao Huang, Jinlian Shao, Jiamin Wu, Xingrou Wu

**Affiliations:** ^1^School of Geography, South China Normal University, Guangzhou, China; ^2^College of Resource Environment and Tourism, Capital Normal University, Beijing, China

**Keywords:** post-traumatic growth, creativity, deliberate rumination, general self-efficacy, mediated effect, moderated mediation model, COVID-19

## Abstract

**Purpose:** This study used a moderated mediation model to test the mediating effect of general self-efficacy on the relationship between post-traumatic growth (PTG) and creativity and the moderating effect of deliberate rumination in the second path of the indirect mediation path during the COVID-19 pandemic.

**Methods:** A sample of 881 university students from Guangdong Province, China, was surveyed with the Posttraumatic Growth Inventory, the Runco Ideational Behavior Scale, the General Self-Efficacy Scale, and the Deliberate Rumination Inventory. SPSS (23 version) and PROCESS (3.3 version) were used for correlation analyses, mediation analysis, and moderated mediation analysis.

**Results:** (1) PTG was positively correlated with creativity, self-efficacy, and deliberate rumination. Creativity was positively correlated with self-efficacy and deliberate rumination. Deliberate rumination was positively correlated with self-efficacy. (2) Self-efficacy mediated the relationship between PTG and creativity. (3) Deliberate rumination moderated the second half of the path of “PTG → self-efficacy → creativity.”

**Conclusions:** PTG affected creativity directly and also indirectly through self-efficacy. In particular, deliberate rumination moderated the relationship between self-efficacy and creativity, such that the association was stronger when the incidence of deliberate rumination was low. These results provide a more comprehensive understanding of the positive link between PTG and creativity.

## Introduction

The coronavirus disease 2019 (COVID-19) spread rapidly and widely worldwide, which had a significant impact on people’s lives (Zhao et al., 2020). For example, a sample survey and analysis of 17,865 posts of active Weibo users found that people’s sensitivity to social risks increased and their life satisfaction decreased during the pandemic (Li et al., 2020). Pandemics and government-mandated measures of quarantine and isolation defined as lockdown have an impact on mental health of general population (Brooks et al., 2020). People suffered from depression, anxiety, insomnia, stress, addiction symptoms, and the persistence of avoidance behaviors due to infection fears, reduced social activities, loss of accessibility to basic necessities, and financial loss during the pandemics (Brooks et al., 2020; Rossi et al., 2020; Grignoli et al., 2021). During the COVID-19 pandemic, a study of 1,210 respondents in China showed that 84.7% spent 20–24 h per day at home and experienced the rates of moderate to severe depression and anxiety of 16.5 and 28.8%, respectively (Wang et al., 2020). In addition, the relatives of COVID-19 patients suffered from high levels of anxiety due to feelings of insecurity and loss of a sense of control (Dorman-Ilan et al., 2020). Most Italian residents had higher levels of distress because of positive cases nearby, the prolonged lockdown and having to relocate (Di Giuseppe et al., 2020). At the same time, people face enormous stressors and spend more time fantasizing, which is an addictive mental behavior associated with psychological dysfunctions (Somer et al., 2020). In summary, individuals have been exposed to varying degrees of threat of injury or death, resulting in numerous psychological problems and instances of post-traumatic stress disorder (PTSD) during the pandemic (Yehuda, 2002; Sun et al., 2020). Therefore, it is essential to understand people’s automatic coping mechanisms in order to respond effectively to the intense stress caused by a pandemic (Di Giuseppe et al., 2020).

The COVID-19 pandemic has also posed a threat to the physical and mental health of Chinese college students, and this has attracted extensive attention from society and academia. Many universities have taken precautionary measures such as closing campuses, postponing internships, and changing to e-learning. College students have generally been anxious because of multiple pressures from the pandemic, their studies and their employment (Cao et al., 2020). For example, a cross-sectional study showed that 50.09% of college students from Guangdong Province, China, reported symptoms of stress (Li X. et al., 2020). A similar study found that the prevalence of mental disorders among 11,954 Chinese college students was as high as 22.8%, and that the stress caused by uncertainty was a risk factor for mental disorders during the pandemic (Wu et al., 2020). In the face of such a devastating public health event, it is necessary to attend to the mental health status of college students and to intervene effectively to improve their ability to avoid mental disorders.

A number of empirical studies have examined the positive effects that traumatic events can have on people (Linley and Joseph, 2004). Tedeschi and Calhoun (1996), in their scientific measurement of the phenomenon of psychological growth after traumatic events, proposed the term *post-traumatic growth* (PTG) to describe the process by which people re-evaluate traumatic events in order to better understand themselves, others, and the world as they actively adapt to challenging circumstances.

Although some college students from China suffered from PTSD, anxiety, and depressive symptoms during the COVID-19 pandemic, they also sought effective ways to adapt to trauma and adversity (Chi et al., 2020). Researchers have pointed out that PTG is positively correlated with PTSD symptoms (Shakespeare-Finch and Lurie-Beck, 2014; Liu et al., 2016) and that both positive and negative outcomes of traumatic events may occur simultaneously. In the short and long post-traumatic period, PTG can be used as a strategy to alleviate PTSD or as an outcome to be transformed by PTSD (Wu et al., 2018). Furthermore, negative emotions and cognitive deficits caused by traumatic events may impair an individual’s creativity development (Benedek et al., 2014). However, a study that focused on 92 outstanding literary scholars from the Tang and Song dynasties in China showed that an adverse environment can be an important factor in the achievement of highly successful creatives (Yi et al., 2017). Trauma and adversity are conceptually similar in that both involve experiences that are destructive or antagonistic to the individual (American Psychiatric Association, 2013). When individuals use various strategies to solve problems in the face of adversity, cognitive flexibility is likely to increase and provide motivation and opportunities for the development of creativity. Then individuals who experience PTG are able to face traumatic events and life challenges with more positive moods, which will foster creative ideas (Han et al., 2019). Thus, trauma is closely linked to creativity, but until now there exists little empirical research examining this association (Liang et al., 2020). In addition, previous studies focused on only outstanding individuals (Simonton, 1994; Niu and Kaufman, 2005), but few studies have investigated the general population. The relationship between ordinary people’s trauma and creativity is also an important issue.

Research shows that the positive changes generated by PTG include interpersonal relationship, personal strength, mental state, attitude toward life, etc. (Tedeschi and Calhoun, 2004). These positive changes can help individuals to adopt more proactive thinking and coping styles when they experience new negative events. Therefore, we speculate that individuals who experience PTG can improve their general self-efficacy. Though the development of creativity may be a manifestation of PTG, it is also influenced by other factors. Studies have demonstrated that individual self-efficacy is a positive predictor of creativity (Jaiswal and Dhar, 2016; Newman et al., 2018). However, it is worth exploring whether self-efficacy can have a positive impact on the creativity of college students in the face of the immense mental stress caused by their social isolation during the COVID-19 outbreak (Chi et al., 2020).

Furthermore, stressors associated with COVID-19 activated individuals’ rumination mechanisms, which may have an impact on individual creativity. It has been shown that a higher frequency of rumination during the pandemic resulted in more negative emotions and decreased cognitive function (Newman et al., 2018; Ye et al., 2020), which is detrimental to the development of creative thinking. However, individuals can also increase their self-confidence by recalling positive examples through rumination (Bandura, 1997). At the same time, creative ideas and material from life can be obtained through rumination (Forgeard et al., 2020). It has been shown that intrusive rumination is positively correlated with creativity (Wang et al., 2021) and that intrusive rumination may promote deliberate rumination (Kamijo and Yukawa, 2018). Deliberate rumination indicates that individuals cognitively revisit events, reframe and recover their core beliefs, and feel positive changes in several aspects of their lives (Cann et al., 2011). When individuals face traumatic events, high self-efficacy can promote individuals to be more likely to actively recognize and reflect on the problem under the influence of the consciousness of “I can,” that is, to realize deliberate rumination (Andersson et al., 2014). However, there are few researches on the influence of deliberate rumination on the relationship between general self-efficacy and creativity (Binnewies et al., 2009). Thus, deliberate rumination deserves attention as an important factor influencing individual mental health and cognitive development.

In order to enhance the understanding of the changes in creativity of college students during the COVID-19 pandemic, this study explored the relationship between PTG and creativity among college students during that period as well as the mediating role of self-efficacy and the moderating role of deliberate rumination. In the following section, the definitions of these four variables and the relationships between them are presented.

## Theoretical Basis and Hypothesis

### Relationship Between PTG and Creativity

PTG refers to the positive psychological changes brought about by an individual’s struggle with traumatic events, which does not mean a reduction in the level of psychological distress; rather, the two often coexist (Tedeschi and Calhoun, 2004). It is commonly assessed using indicators such as “new possibilities, relating to others, personal strength, spiritual change, and appreciation of life” (Tedeschi and Calhoun, 1996). Recently, many studies have focused on the predictors of and influences on PTG. For example, it has been shown that optimism, social support, and appropriate cognitive strategies contribute to PTG (Baillie et al., 2014; Pérez-San-Gregorio et al., 2017; Sun et al., 2020). In addition, a number of scholars and practitioners have empirically confirmed the mechanisms of action when different traumatic events have a positive impact on people such as cancer (Sharp et al., 2018), burns (Wiechman Askay and Magyar-Russell, 2009), sexual assault (Frazier et al., 2001), earthquakes (Alamdar et al., 2020), and the death of a loved one (Büchi et al., 2007). Although the possible positive effects of traumatic events have been demonstrated in practice, the exact nature of the predictors and consequences of PTG remain inconclusive (Blackie and Jayawickreme, 2014; Groarke et al., 2017).

Guilford (1950) defined the term *creativity* as a process of thought and action that produces new and original works, emphasizing the originality and effectiveness of creativity (Runco and Jaeger, 2012). Rhodes (1961) proposed the *4Ps creativity model*, which integrates the different aspects of creativity, including person, process, product and process. In the field of social psychological research, Amabile (1988) developed a model of the components of creativity that comprised cognitive, personality, motivational, and social factors including domain-relevant skills, creativity-relevant skills and task motivation. Later, Amabile (1996) revised the model to take into account the working environment. Sternberg and Lubart (1991) developed *creativity investment theory*, which states that creativity is related to six factors associated with the individual’s psychological mechanisms and environment: intellectual abilities, knowledge, thinking styles, personality, motivation, and environment. Plucker et al. (2004, p. 90), after reviewing 90 articles from high-impact journals, suggested that “creativity is the interaction among aptitude, process, and environment by which an individual or group produces a perceptible product that is both novel and useful as defined within a social context.” However, there is currently no agreed definition of creativity (Treffinger, 2009).

Creativity is an important part of cognitive, social, and emotional activity, and it is often defined in terms of creative products, but creative ideas can also be quantified as creative products, and focusing on the ideas people generate is particularly useful for understanding “everyday creativity” (Runco et al., 2000). The Runco Ideational Behavior Scale, for example, measures creative potential by asking individuals to rate the frequency with which they generate ideas in their everyday experiences (Runco et al., 2000).

A number of studies have examined the influences on an individual’s creativity such as environment, emotions, cognition, stress, goals and motivation (Peterson et al., 2008; Edl et al., 2014; Hu et al., 2015; Du et al., 2020). Peterson et al. (2008) found that the character strength of creativity correlated significantly with PTG (Peterson et al., 2008). It is a pity that creativity was only one of many character strengths included as outcomes in their study, the precise nature of the association between PTG and creativity was not investigated further or explained.

Creative processes involve cognition and self-control (Edl et al., 2014). Traumatic events may impair cognitive processes and cognitive functioning, thus negatively affecting creativity (Paulus et al., 2010). Conversely, an examination of the experiences of 722 Chinese writers in the twentieth century found that those who suffered personal tragedy or political persecution were more likely to win creative awards in their later years (Niu and Kaufman, 2005). Thus, the effects of trauma can be both negative and positive.

PTG can be accompanied by positive psychological changes, i.e., in personal attitudes, awareness, and health behaviors associated with growth (Siegel and Schrimshaw, 2000; Barskova and Oesterreich, 2009). It has been shown that increased creativity may constitute a manifestation of PTG (Forgeard, 2013). During the COVID-19 outbreak, Chinese university students have shown the ability to respond effectively to challenges and experience PTG (Chi et al., 2020). However, the positive changes in the behavior and the cognition of college students experiencing PTG need to be further explored through empirical studies (Liang et al., 2020). In summary, we believe that PTG can have a positive effect on the creativity of college students and propose the following hypothesis:

*H1*: PTG has a positive correlation with creativity.

### The Mediating Role of Self-Efficacy

*Self-efficacy* is a central concept in social cognitive theory (Bandura, 1978), which refers to an individual’s perceptions or beliefs about whether they are able to adopt appropriate behavior when faced with challenging circumstances. As a perception of “can do,” self-efficacy reflects a sense of control over the environment and may be thought of as the ability of individuals to handle certain life stressors with greater confidence (Schwarzer et al., 1997). According to the theory of self-efficacy, the sense of the individual of control over the environment and subjective evaluation of their own ability will affect their psychological status and behavioral choices. These include what behavioral choices to make, how much effort to put in and how long to persist in the face of difficulties, and the emotional state of the person facing the situation (Bandura, 1990). Self-efficacy is not static (Ng and Lucianetti, 2015), and it is influenced by a variety of internal and external factors such as achievement goals (Du et al., 2020), creativity training (Mathisen and Brønnick, 2009), and social support (Mathisen, 2011).

Previous research has shown that PTG is positively correlated with self-efficacy in both patient and survivor groups that have experienced a traumatic event (Barskova and Oesterreich, 2009). Self-efficacy manages people’s perceptions of the environment and their assessment of personal competence in the face of traumatic and stressful events (Bandura, 2011). People with high self-efficacy are more open to challenges thus are more likely to experience growth-related changes. PTG, both as a process and as an outcome, also can impact positively on an individual’s personality (Blackie and Jayawickreme, 2014). Therefore, we speculate that individuals who experience traumatic events and achieve growth can improve their general self-efficacy. Thus, it is proposed that individuals who experience PTG are more confident in themselves and, in their lives, then experience greater general self-efficacy in the face of stressful events (Tedeschi and Calhoun, 2004; Jia et al., 2017). It leads to the second hypothesis of the study:

*H2a*: PTG has a positive correlation with self-efficacy.

A number of studies have investigated the relationship between self-efficacy and creativity. In the field of creativity research, the positive correlation between an individual’s self-efficacy and an individual’s creativity is well recognized (Jaiswal and Dhar, 2016; Newman et al., 2018). In addition, some studies have explored the mediating role of self-efficacy in the relationship between creativity and other factors such as achievement goals (Du et al., 2020), motivation-enhancing practices (Ma et al., 2017), and active procrastination (Liu et al., 2017). The predictive effect of self-efficacy on creativity is also influenced by internal and external factors. For example, self-efficacy had a differential effect due to individual differences in creativity, and that it was negatively correlated with individual creativity for employees who were more promotion-oriented (Li C.-R. et al., 2020). In addition, researchers have empirically shown that college students’ award experience influences the degree of effect of their self-efficacy on creativity (Chang et al., 2016). Given so much overlap between self-efficacy and creativity, we propose the third hypothesis:

*H2b*: Self-efficacy has a positive correlation with creativity.

From a health behavior perspective, self-efficacy affects people’s health behaviors (Strecher et al., 1986). For example, the person who has high sense of self-efficacy is more likely to increase motivation to act, leading to greater achievement and better health (Zalewska-Puchala et al., 2007). This means that individuals who have increased self-efficacy during a traumatic event are likely to experience more positive changes. Thus, they are able to confidently cope with illness risks and implement positive and healthy behaviors, thereby promoting creative behaviors. In addition, self-efficacy mediates knowledge and behavior and facilitates the knowledge-behavior relations (Rimal, 2000). It has also been shown that self-efficacy mediates perceived efficacy of the government health measures and compliance during the pandemic, and that people with high self-efficacy are better able to have higher scores in behavioral compliance (Roma et al., 2020). It follows that self-efficacy as a factor that enables individuals to face life stresses with confidence is often presented as a mediating role in research.

Individuals who have experienced PTG may develop confidence in their own abilities and in their capacity to face the future, which may facilitate their achievement of more challenging and creative tasks. The COVID-19 has forced college student to study at home for long periods of time, resulting in students facing multiple stressors and suffering from general anxiety about their physical health, academics, and socialization (Cao et al., 2020; Li X. et al., 2020). Thus, COVID-19 has been a substantial stressor that can lead to psychological distress among college students (Lahav, 2020). Exposure to stressors leads to the secretion of cortisol in the body (Dickerson and Kemeny, 2004). It has been shown that physiological activity in humans is influenced by self-efficacy and that cortisol secretion is lower at when self-efficacy is high (Wiedenfeld et al., 1990, Nierop et al., 2008), thereby reducing the increase in cortisol that may result from traumatic events and mitigating the potentially damaging effects on neuronal and cognitive mechanisms (Schönfeld et al., 2013). This physiological mechanism reveals that an individual’s self-efficacy may have an effect on creative cognition by influencing the secretion of cortisol. Thus, the increase in self-efficacy, as an important aspect of PTG, protects psychological health, which facilitates the generation of creative ideas. On the other hand, changes in self-efficacy, an important judgment factor in the choice of challenging tasks and problem-solving, may have an impact on creativity. Then, we propose the fourth hypothesis.

*H2*: Self-efficacy mediates PTG and creativity.

### The Moderating Role of Deliberate Rumination

There are two main types of definitions of rumination in current research. Nolen-Hoeksema et al. (2008) defined *rumination* as an individual’s repeated, passive attention to the details of a stressful event, the possible causes and consequences of its symptoms, and the details of the course of the event. However, to assess contemplation objectively and neutrally, it is important to distinguish two main types of rumination: intrusive and deliberate (Tedeschi and Calhoun, 2004; Cann et al., 2011). *Intrusive rumination* refers to the individual’s passive repetition of the traumatic event in a negative manner, which is a non-constructive cognitive approach, whereas *deliberate rumination* refers to the individual’s active re-examination of the traumatic events and related information: they face the dilemma openly and solve the problem (Cann et al., 2011). Deliberate rumination represents the positive aspect of contemplation, whereby individuals actively reflect on and re-evaluate cognitive processes and ways of thinking to choose a more compatible worldview and lifestyle, shifting their personal attention to positive aspects (Joseph and Linley, 2005). Deliberate rumination allows individuals to achieve positive meaning construction of the stressful event (Cann et al., 2010b), which may lead to PTG of the individual (Wu et al., 2015). What’s more, deliberate rumination can lead to a more purposeful cognitive process and metacognition of the stressful event, which aims to solve problems and foreshadows the possibility of PTG (Cann et al., 2011), reducing the damage of the stressful event (Tedeschi and Calhoun, 2004). For example, in a study of PTG following cancer, it was found that enhanced deliberate rumination can facilitate individuals to achieve PTG (Rider Mundey et al., 2018). Studies have found a significant positive correlation between deliberate rumination and PTG (Andrades et al., 2017; Xu et al., 2019). From above, we can infer that, to some extent, deliberate rumination seems to be an important factor in the individual’s perception of a potentially negative stressor. In summary, we propose a fifth hypothesis:

*H3a*: Deliberate rumination has a positive correlation with PTG.

Studies examining the effects of rumination on creativity have shown that different types of rumination affect creativity differently. An increase in creative thinking may stem from the development of a post-traumatic cognitive process (Liang et al., 2020), which involves cognitive and attentional control. Iterative rumination on individuals and events is also an important factor in the development of creativity (Verhaeghen et al., 2005). For example, it has been suggested that rumination contributes to the development of higher-value creative ideas (Forgeard et al., 2020). Self-reflective rumination stimulates interest in creative behavior (Verhaeghen et al., 2005, 2014). In light of the above, we believe it is worthwhile to further explore how deliberate rumination can contribute to the development of creativity in college students. Therefore, we hypothesize that.

*H3b*: Deliberate rumination has a positive correlation with creativity.

The relationship between self-efficacy and rumination has been explored from different perspectives. Researchers have shown that rumination has a negative impact. For example, an empirical study with undergraduate nursing students showed that rumination can lead to depression and reduce self-efficacy, and that self-efficacy does not alleviate depression (Takagishi et al., 2013).

Conversely, higher levels of self-efficacy contribute to an individual’s ability to adapt and develop. Individuals with PTSD and possessing higher self-efficacy chose to extract memories that promoted an increase in self-efficacy when recalling experiences (Brown et al., 2016). In other words, self-efficacy had a positive impact on deliberate rumination. It is generally accepted that rumination is likely to trigger negative emotions and thoughts in individuals, but the facilitative role of rumination with positive beliefs in problem-solving has been overlooked (Dunn and Sensky, 2018). Furthermore, finding support for one’s view of oneself by reviewing the past and constructing future events is often beneficial in establishing and maintaining self-efficacy (Bandura, 1997). Therefore, we suggest that there is an association between self-efficacy, deliberate rumination and creativity, and that, due to its plasticity (Ng and Lucianetti, 2015), self-efficacy has different effects on creativity at different levels of deliberate rumination. In summary, we hypothesize that.

*H3c*: Deliberate rumination has a positive correlation with self-efficacy.

Based on existing research and the hypotheses above, we further propose that

*H3*: Deliberate rumination moderates the second half of the path of “PTG → self-efficacy → creativity.”

## Materials and Methods

### Participants and Procedures

The study was conducted at a polytechnic in Guangdong Province, China, that serves more than 20,000 full-time students. A total of 918 students completed the survey questionnaire. After data collection, 37 participants who were not from Guangdong were excluded from the study, and so the actual number of valid questionnaires was 881. Among the interviewees, 317 (35.982%) were male and 564 (64.018%) were female. Before the research design was finalized, the researchers conducted exploratory focus-group interviews with students at the school to understand their emotional profile and psychological state. The majority of the interviewees indicated that they had been depressed during the COVID-19 pandemic.

The present study followed a correlational design and used a web-based questionnaire as the data collection method. The questionnaires were completed between April 10 and June 15, 2020. A QR code for completing the questionnaire was sent to students electronically during the time college students were studying online because their in-person classes were canceled because of the COVID-19 outbreak. Participants simply had to scan the QR code, go to the on-screen questionnaire, answer the questions and then click on Submit (QR refers to *quick response*, a readable barcode that contains a large amount of information. Devices, such as mobile phones and tablets, use cameras to scan the QR code and recognize the binary data in it, which takes them to a specific linked website). In China, QR codes are widely used as a means of accessing specific webpages and for other tasks, such as making financial payments, providing identification and searching for information. It should be emphasized that the purpose of the study was explained in detail before the QR code was scanned and that all participants completed the questionnaire on a voluntary basis.

### Materials

The questionnaire used in this study consisted of 64 items divided into five sections: (a) demographic information, (b) Posttraumatic Growth Inventory, (c) Runco Ideational Behavior Scale, (d) General Self-Efficacy Scale, and (e) Deliberate Rumination Inventory. The demographic information included gender, home address, and profession. Runco Ideational Behavior Scale, General Self-Efficacy Scale, and Deliberate Rumination Inventory above-mentioned scales were originally developed in English and translated into Chinese for this study. In order to improve the quality of the translations, a back-translation method was used (Brislin, 1970); that is, the first researcher translated the English version into Chinese, then a second researcher back-translated the translated English into Chinese, and a third researcher compared the original, translated, and back-translated versions of the scales to assess the accuracy of the translations. The translations were corrected and optimized prior to finalizing the questionnaire to ensure the equivalence of the scales.

#### Posttraumatic Growth Inventory

The study used the Chinese version of the Posttraumatic Growth Scale, originally proposed by Tedeschi and Calhoun (1996) and later translated by Geng et al. (2011). The scale consists of 21 items that cover five dimensions: interpersonal relationships (e.g., “Putting effort into my relationships”), new possibilities (e.g., “I’m more likely to try to change things which need changing”), personal strengths (e.g., “I discovered that I’m stronger than I thought I was”), spiritual changes (e.g., “A better understanding of spiritual matters”), and the appreciation of life (e.g., “Appreciating each day”). The scale has six points that measure feelings, reactions, and agreement (1 = no change to 6 = very high degree of change). In the present study, the Cronbach’s alpha coefficient of the scale was 0.958.

#### Runco Ideational Behavior Scale

The Runco Ideational Behavior Scale, developed by Runco et al. (2000), was used in this study. It consists of 23 self-report items (e.g., “I think about ideas more often than most people” and “I am able to think up answers to problems that have not already been figured out”) that measure the level of creative behavior in everyday life on a 5-point Likert scale (1 = strongly disagree to 5 = strongly agree). In the present study, the Cronbach’s alpha coefficient of the scale was 0.938.

#### General Self-Efficacy Scale

The General Self-Efficacy Scale (Schwarzer et al., 1997), which consists of 10 items, was used in this study. After discussion, the last three items of the original scale were removed so as to take into account the specific situation of participants and research needs, reducing the total to seven (e.g., “I can always manage to solve difficult problems if I try hard enough” and “I am confident that I could deal efficiently with unexpected events”). The scale has four points (1 = not at all true to 4 = very true). In this study, the Cronbach’s alpha coefficient of the scale was 0.875.

#### Deliberate Rumination Inventory

The Deliberate Rumination Inventory, part of the Event-Related Rumination Inventory, developed by Cann et al. (2011), was used in this study. The inventory, consisting of 10 items (e.g., “I thought about whether I could find meaning from my experience” and “I thought about the event and tried to understand what happened”), assesses the frequency of deliberate rumination in injured people. The scale rated on a 4-point Likert scale (1 = not at all and 4 = always). In this study, the Cronbach’s alpha coefficient for the inventory was 0.913.

### Data Analysis

Version 23.0 of SPSS was used to perform the analysis. Since self-report data were collected for this study, common method biases were tested to ensure its validity by using the Harman single factor test before data processing (Podsakoff et al., 2003). The 74 items in the questionnaire related to the four variables were tested. The results showed that nine factors had eigenvalues greater than 1. These factors contributed 63.876% of the total variance. The first factor explained only 31.878% of the variance, which did not reach the critical criterion of 40% (Zhou and Long, 2004), indicating that there is no significant common methodological bias in this study.

After common-method-bias evaluation, we carried out descriptive analysis, correlation analysis, and model testing of the data based on the research hypotheses. First, we examined trends in concentration and dispersion of the data. Then, we tested the relationships between the independent, mediating, dependent, and moderating variables by calculating Pearson’s correlation coefficients. A model was constructed based on the results of the correlation analysis, and the hypotheses proposed were tested, and the PROCESS (version 3.3) plug-in in SPSS was used to test the mediating effect of self-efficacy and the moderating effect of deliberate rumination. [The PROCESS plug-in was developed by Hayes (2013) specifically for path analysis-based regulation and mediator analysis and their combinations].

## Results

### Descriptive Statistics and Correlations Analyses

Means, standard deviations, and correlations of the study variables were calculated. As shown in Table 1, the PTG of participating college students was significantly and positively correlated with general self-efficacy (*r* = 0.466, *p* < 0.01) and creativity (*r* = 0.434, *p* < 0.01). Their general self-efficacy was positively correlated with creativity (*r* = 0.475, *p* < 0.01) and deliberate rumination (*r* = 0.216, *p* < 0.01). In addition, deliberate rumination was positively correlated with creativity (*r* = 0.288, *p* < 0.01). Thus, the results of the correlation analysis provided preliminary support for the subsequent mediated-effects test. In addition, gender was used as a control variable in the current study. And, it was dummy coded (1, female and 0, male).

**Table 1:** Descriptive statistics and correlations among variables.

Variables	Mean	SD	1	2	3	4
(1) PTG	3.300	1.003	—			
(2) Creativity	3.249	0.563	0.434^**^	—		
(3) GES	2.318	0.537	0.466^**^	0.475^**^	—	
(4) DR	2.012	0.520	0.353^**^	0.288^**^	0.216^**^	—

*N* = 881. PTG, post-traumatic growth; GES, general self-efficacy; and DR, deliberate rumination.

** *p* < 0.01;

*** *p* < 0.001.

### Mediation Analysis of Self-Efficacy

Multiple regression analysis was performed using Model 4 of the PROCESS component of SPSS, with PTG as the independent variable, creativity as the dependent variable and general self-efficacy as the mediating variable. As shown in Table 2, PTG positively predicted creativity (*β* = 0.150, SE = 0.018, *p* < 0.001). As well, PTG was positively correlated to self-efficacy (*β* = 0.248, SE = 0.016, *p* < 0.001), and self-efficacy was positively correlated to creativity (*β* = 0.375, SE = 0.035, *p* < 0.001).

**Table 2:** Testing the mediating effect of PTG on creativity.

Predictors	On GSE				On creativity			
	*β*	SE	*t*	95% CI	*β*	SE	*t*	95% CI
Gender	−0.199	0.033	−6.096 ^***^	[−0.264, −0.135]	0.044	0.034	1.283	[−0.023,0.111]
PTG	0.248	0.016	15.820^***^	[0.217,0.279]	0.150	0.018	8.270^***^	[0.115,0.186]
GES					0.375	0.035	10.843^***^	[0.307,0.443]
*R*^2^	0.249				0.285			
F	149.807^***^				116.346^***^			

Analyses conducted using PROCESS model 4. *N* = 881.

PTG, post-traumatic growth and GES, general self-efficacy.

** *p* < 0.01;

*** *p* < 0.001.

In addition, we used the bootstrap method to test the confidence interval (CI) estimates, which showed that the 95% confidence intervals for the direct and indirect effects of PTG on creativity did not include 0. Thus, the partial mediator equation model of general self-efficacy held, and self-efficacy was the mediating variable in the relationship between PTG and creativity. The direct effect (0.150) and the mediating effect (0.093) accounted for 61.728 and 38.272% of the total effect, respectively (see Table 3).

**Table 3:** Total effect, direct effect and indirect effect among the variables.

	Effect size	Boot SE	Boot CI lower limit	Boot CI upper limit	Relative effect size
Total effect	0.243	0.017	0.210	0.277	
Direct effect	0.150	0.018	0.115	0.186	61.728%
Indirect effect	0.093	0.012	0.070	0.118	38.272%

### Moderated Mediation Effects

**Figure 1:**
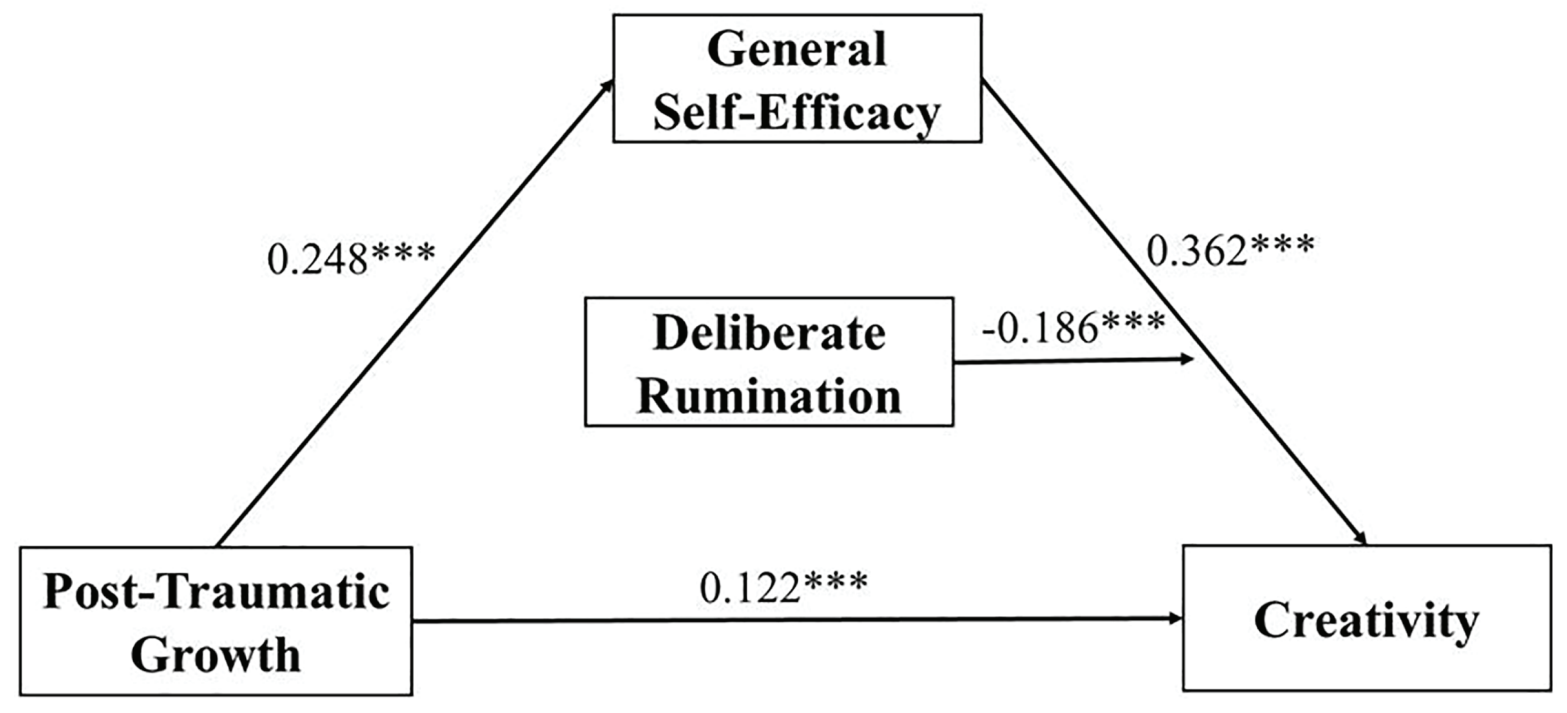
The moderated mediation model.

To test H3, the second half of the mediated model was analyzed by adding the moderating variable deliberate rumination. Then we used SPSS PROCESS Model 14 to test the model. The results showed that deliberate rumination was positively correlated to creativity (*β* = 0.178, *p* < 0.001) with a 95% CI [0.111, 0.244]. The interaction term of self-efficacy and deliberate rumination reached a significant level for creativity (*β* = −0.186, *p* < 0.001) with a 95% confidence interval of [−0.282, −0.090] (see Table 4). The model is shown in Figure 1.

**Table 4:** Testing the moderated mediating effect of PTG on creativity.

Predictors	On GSE				On creativity			
	*β*	SE	*t*	95% CI	*β*	SE	*t*	95% CI
Gender	−0.199	0.033	−6.096^***^	[−0.264, −0.135]	0.021	0.034	0.613	[−0.046,0.087]
PTG	0.248	0.016	15.820 ^***^	[0.217,0.279]	0.122	0.019	6.553^***^	[0.086,0.159]
GES					0.362	0.034	10.626^***^	[0.295,0.429]
DR					0.178	0.034	5.222^***^	[0.111,0.244]
GSE*DR					−0.186	0.049	−3.814^***^	[−0.282,-0.090]
*R*^2^	0.249				0.311			
F	145.807^***^				79.000^***^			

Analyses conducted using PROCESS model 14. *N* = 881.

PTG, post-traumatic growth; GES, general self-efficacy; and DR, deliberate rumination.

** *p* < 0.01;

*** *p* < 0.001.

**Figure 2:**
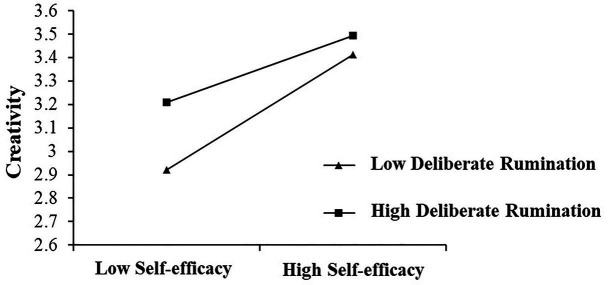
The relationship between self-efficacy and creativity for high and low levels of deliberate rumination.

In order to further analyze the moderating effect of deliberate rumination, the study divided deliberate rumination into low (M − 1SD) and high (M + 1SD) groups and performed a simple slope analysis. The results showed that 95% of the CIs did not include zero and that deliberate rumination influenced the strength of the relationship between self-efficacy and creativity (see Table 5). Self-efficacy was a stronger predictor of creativity with low (i.e., M − 1 SD) levels of deliberate rumination (see Figure 2).

**Table 5:** Conditional indirect effect at specific levels of deliberate rumination when mediated by general self-efficacy.

Conditional effect of DR	Effect	Boot SE	95%CI
Low (M – 1 SD)	0.114	0.016	[0.081,0.147]
Medium (M)	0.090	0.012	[0.066,0.114]
High (M + 1 SD)	0.066	0.015	[0.036,0.094]

## Discussion

### Discussion of the Results

First, the present study shows that PTG significantly predicts creativity in college students, which was relatively consistent with H1 and previous studies on the positive effects of traumatic events, which have the potential to increase creativity (Forgeard, 2013; Liang et al., 2020). According to the PTG model proposed by Tedeschi and Calhoun (2004), traumatic events change an individual’s worldview, and PTG is the result of reconstruction and integration of cognitive assessments of traumatic events. The cognitive assessment process may involve cognitive neurological and psychological rehabilitation, prompting reflection and return to a way of life more in line with their values, thus changing their behavior and thinking (Baseotto et al., 2020). At the same time, from a mental and behavioral developmental perspective, negative experiences can have an impact on an individual’s cognitive and behavioral structure (Yi et al., 2017). When faced with difficulties and distress, they think deeply and explore ways to find a way out of the situation. In the process, they choose new ways of thinking and make an effort to adopt behaviors that build new cognitive structures and behavioral systems (Helson and Roberts, 1994). By using a variety of strategies to solve problems and readjust to a new life, they successfully embrace new ways of making sense of the world and develop new cognitive structures that promote cognitive flexibility and innovative behavior (Cann et al., 2010a). As a result, individuals who have experienced PTG have a more objective and positive perception of the traumatic event and on the challenges of everyday life. This positive emotional state is conducive to creative development (Han et al., 2019). So, measuring levels of PTG can predict an individual’s perception of their increased level of creativity.

Second, the findings validate H2a, H2b, and H2. The results show that PTG positively predicts an individual’s self-efficacy, which aligns with previous findings that individuals who have experienced PTG may be able to develop greater confidence in themselves (Joseph et al., 1993; Arpawong et al., 2013; Glad et al., 2013). This finding supports the PTG model, which posits positive changes in self-perception (Tedeschi and Calhoun, 2004). In other words, the positive impact of a traumatic event may also be reflected in the individual’s reassessment of their ability to face and resolve past or possible future traumatic events in a more confidently and courageously. In addition, individuals with high self-efficacy are more likely to experience PTG (Benight and Bandura, 2004; Prati and Pietrantoni, 2009; Jurišová, 2016). Thus, PTG and self-efficacy are closely related.

The results also validate the positive predictive effect of self-efficacy on creativity, which also aligns with the results of previous studies. According to social cognitive theory, people with high self-efficacy tend to be more open to challenges, put a higher level of effort into an activity and pay more sustained attention to it (Bandura, 1978, 1982). Therefore, with regard to creativity, self-efficacy affects the individual’s abilities to engage in the creative process: persons with low self-efficacy may cease their efforts when faced with challenges and dilemmas, and so fail to produce a product of creative value. In addition, the component model of creativity points by Amabile (1988) to internal motivation as one of the most important factors influencing creativity. Individuals with high self-efficacy, which is a source of motivation, tend to set creative goals and are confident of reaching them (Intasao and Hao, 2018). However, those with doubts about their own abilities tend to avoid situations and tasks that are beyond their creative reach, which can make it difficult for them to develop and demonstrate practical skills and abilities (Locke and Latham, 2006). During the COVID-19 pandemic, college students who experienced PTG may have increase their level of self-efficacy, which had a protective effect on cognitive (Wang et al., 2014). As a result, they have been able to gather materials and ideas from life in a more positive frame of mind and stimulate their creative behavior. In summary, it is logical that self-efficacy plays a mediating role between PTG and creativity.

Third, the results are also consistent with H3a, H3b, H3c, and the results of other studies. To begin, deliberate rumination by college students had a positive impact on PTG, which validates previous research that showed that deliberate rumination has a constructive effect on human development. Therefore, in mental health counseling and interventions for college students, it is important to help them to transform cathartic negative thoughts and emotions into positive perceptions of the traumatic event in order to facilitate the occurrence of PTG. Further, deliberate rumination had a positive effect on college students’ creativity (Verhaeghen et al., 2005, 2014; Forgeard et al., 2020). In past studies, self-efficacy and rumination were considered as important factors influencing individual physical and mental growth, with the former often representing a positive influence and the latter being associated with mental illness (Takagishi et al., 2013; Karabati et al., 2017; Nota and Coles, 2018), However, the two dimensions of rumination have not been comprehensively discussed. Deliberate rumination, which involves active self-reflection and reflection, can help individuals to better cope with difficult situations (Dunn and Sensky, 2018). Finally, deliberate rumination had a positive correlation with students’ self-efficacy, which is consistent with the findings of previous studies (Bandura, 1997; Brown et al., 2016). The frequency of rumination among college students influenced their level of creativity and deliberate rumination, which means individual have positive beliefs stimulates creative interests and behaviors.

Fourth, the present study found that deliberate rumination plays a moderating role in the influence of college students’ self-efficacy on creativity, which confirms H3. The present study found that the positive effect of self-efficacy on creativity was more significant for college students with lower levels of deliberate rumination compared to those with high levels of deliberate rumination. According to Cann et al. (2011), deliberate rumination enables people to positively understand negative events in order to solve problems. As mentioned above, deliberate rumination has been found to be an important predictor of PTG. For example, it has been shown that deliberate rumination motivates individuals to rethink the world, others, and self and to take the initiative to obtain social support to reduce stress and grow (Xu et al., 2019). Therefore, college students with higher levels of deliberate rumination have the ability and means to solve problems and rate their level of creativity higher. In contrast, students with low levels of deliberate rumination inhibit the development of creativity due to a lack of proactive and constructive thinking about events and problems (Cohen and Ferrari, 2010; Vahle-Hinz et al., 2017).

However, it does not mean that higher levels of deliberate rumination are better, because students with low levels of deliberate rumination were more likely to be influenced by self-efficacy as a factor. As the frequency of deliberate rumination decreased, the effect of self-efficacy on creativity increased. While deliberate rumination assists individuals in finding meaning in stressful events, this meaning may involve negative beliefs, worldviews, and self-concepts (Kamijo and Yukawa, 2018), which may lead to negative emotions, which reveals that when suffer a stressful event, individuals who possess strong deliberate rumination may reinforce the recurrence of negative emotions to the extent that they reduce their self-efficacy in dealing with the stressful event, thus weakening the development of creativity. The high levels of deliberate rumination are more likely lead to negative emotions due to the fact that during the COVID-19 outbreak, students studied at home for long periods of time and were unable to communicate with others and so resolve their confusion. Then changes in self-efficacy have less impact on the effect of creativity under the effect of negative emotions such as anxiety and helplessness (Hu et al., 2015). Dispersed thinking is a core component of creative thinking (Runco and Acar, 2012). When deliberate rumination levels are lower, individuals with high levels of self-efficacy are able to positively think and solve problems from multiple perspectives due to reduced frequency of repetitive thinking about events and constant self-focusing. Then the influence of self-efficacy on creativity is subsequently enhanced.

### Implications

In a theoretical sense, our study links PTG and creativity, a new learning that deepens understanding of the positive impact of traumatic events on the mechanisms of creativity. In addition, the researchers analyzed mediating and moderating effects. Using self-efficacy as a mediating variable, they found that the PTG of college students increased their self-efficacy and ultimately had a positive effect on their creativity, while deliberate rumination had a moderating effect on self-efficacy and creativity.

In a practical sense, this study has described the relationship between the four variables presented in this study, which may help researchers to better understand the mechanisms by which creativity develops after trauma. Therefore, the negative impact of major public health events, such as the COVID-19 pandemic, can be alleviated through positive psychological interventions with the college student population. This theory has a great guiding role in reality. After college students experience traumatic events, effective measures can be taken to promote their PTG, improving their self-efficacy and avoiding them having too high frequency of deliberate rumination so as to develop their creativity. During the outbreak confinement, The Ministry of Education of the People’s Republic of China asked universities to set up psychological support hotlines and online counseling services to mitigate the psychological damage caused to students. For instance, several universities provided psychological counseling services for students, offering them access to psychological problems, timely follow-up by professionals, and regular visits thus providing better attention to students’ PTG (The Ministry of Education of the People’s Republic of China, 2020). Second, news media should also focus on promoting healthy lifestyles and new forms of activities that emerge during the COVID-19 pandemic, such as online supermarkets, video consultations, and live home activities, which will help the public to build confidence in their lives and avoid excessive attention of epidemic-related information. Meanwhile, the mutual assistance of epidemic prevention resources and the timely response of staff to residents’ needs in the community will enhance individuals’ sense of security and alleviate the negative feelings associated with isolation and excessive rumination. Such interventions can help the students to reconstruct their perception of adversity and enable them to develop positive self-beliefs and so promote the creative process.

### Limitations and Future Directions

This study has certain limitations. First, the cross-sectional study used in this study, while revealing correlations between the variables, does not allow for inference of causality between the variables tested. Future researchers could conduct longitudinal studies to determine whether the scores measured effectively represent an actual increase in creativity. Second, this study was conducted with college students from the same college, and due to the limitations of sampling at that time, gender balance of the samples was not achieved. In the future, the external validity of the results of this study can be tested by selecting subjects from a wider range of sources and more balanced gender. Third, this study only focused on the moderating effect of deliberate rumination between self-efficacy and creativity. Future research could further expand on the effects of other dimensions of rumination on creativity after a traumatic event. Finally, the effects of other factors, such as major discipline and gender on the four variables, have not been explored in this study, and future research could include control variables to further clarify the relationship between the four variables.

## Conclusion

This study tested a moderated mediation model to examine the relationship between PTG and creativity, and the mediating role of self-efficacy between the two and the moderating role of deliberate rumination in college students during the COVID-19 pandemic. The results showed that PTG positively predicted creativity while self-efficacy mediated the relationship between the two. Furthermore, deliberate rumination moderated self-efficacy in the second half of the mediating pathway between PTG and creativity. More specifically, the positive predictive effect of self-efficacy on creativity was more pronounced at low levels of deliberate rumination.

## Data Availability Statement

The raw data supporting the conclusions of this article will be made available by the authors, without undue reservation.

## Ethics Statement

The studies involving human participants were reviewed and approved by the Institutional Ethics Committee of School of Geography, South China Normal University. The patients/participants provided their written informed consent to participate in this study.

## Author Contributions

WZ and YX designed the research and reviewed and edited the paper. YZ, WZ, YX, and DH carried out the literature search and data analysis. WZ, YZ, YX, JS, XW, JW, and DH wrote the paper. All authors have read and agreed to the published version of the manuscript.

### Conflict of Interest

The authors declare that the research was conducted in the absence of any commercial or financial relationships that could be construed as a potential conflict of interest.
